# Editorial: Linking cellular metabolism to hematological malignancies, volume II

**DOI:** 10.3389/fonc.2024.1387249

**Published:** 2024-04-09

**Authors:** Zhuojun Liu, Jian Yu, Zhizhuang Joe Zhao, Hubing Shi, Manoj Kumar Kashyap

**Affiliations:** ^1^ Key Laboratory of Biomechanics and Mechanobiology, Beijing Advanced Innovation Center for Biomedical Engineering, School of Biological Science and Medical Engineering, Beihang University, Beijing, China; ^2^ Department of Pathology, University of Oklahoma Health Sciences Center, Oklahoma City, OK, United States; ^3^ Laboratory of Integrative Medicine, Clinical Research Center for Breast, State Key Laboratory of Biotherapy, West China Hospital, Sichuan University and Collaborative Innovation Center, Chengdu, Sichuan, China; ^4^ Amity Stem Cell Institute, Amity Medical School, Amity University Haryana, Gurugram, Haryana, India

**Keywords:** ferroptosis, multiple myeloma, lipid reprogramming, RNA-Seq, Reactive oxygen active (ROS), leukemia

Cancer cells reprogram their metabolism to survive in hostile environments, which may reveal potential vulnerabilities to targeted therapies. Metabolic-based therapies can specifically target cancer metabolism, resulting in substantial antitumor effects, while sparing normal cells. This Research Topic highlights the importance of understanding tumor metabolic remodeling and immunometabolism in hematological malignancies.

Rana et al. studied cell metabolism changes in multiple myeloma. Cancer cells use aerobic glycolysis for energy, unlike healthy cells that use mitochondrial oxidative phosphorylation. This shift helps cancer cells obtain nucleotides, amino acids, and lipids for replication. Proteasomes, which regulate protein levels affecting metabolism, survival, and growth, are crucial in both healthy and cancerous cells. Proteasome inhibitors, developed over the past two decades, have improved patient survival and quality of life in multiple myeloma.

Ferroptosis is a recently discovered, iron-dependent cell death process. This leads to Oxidative stress and cell death occur due to the accumulation of lipid reactive oxygen species (ROS). Liu et al. revealed that blood tumor cells, such as leukemia and lymphoma cells, are sensitive to ferroptosis.

Lipid metabolism and hematological malignancies have a complex relationship, presenting the challenges and opportunities for therapeutic approaches. Lipid reprogramming is crucial for tumor cell physiology and influences cellular functions are necessary for cancer growth and survival. Zhang et al. highlighted the complexity arising from the interconnectedness of glucose, lipid, and amino acid metabolism within cancer cells, describing how these metabolic pathways affect and regulate each other in intricate ways, creating challenges in effectively inhibiting cancer growth by targeting a single metabolic process alone.

L-Asparagine (L-ASNase) is a hydrolytic enzyme that reduces circulating asparagine, a crucial amino acid for the survival and growth of leukemia cells, particularly lymphoblasts in acute lymphocytic leukemia (ALL). As leukemic cells lack the enzyme asparagine synthetase, they depend on external asparagine sources. By starving them of this essential amino acid, L-ASNase selectively targets and kills leukemia cells, while sparing normal cells that can synthesize asparagine. It has been widely used to treat leukemia, including childhood leukemia. Zhou et al. summarized the different mechanisms of drug resistance to L-ASNase. L-ASNase immunogenicity can trigger the production of antidrug antibodies that neutralize L-ASNase activity by binding to this preventing its action on leukemia cells. As a result, the drug is cleared from the bloodstream faster, thereby reducing its therapeutic effects. Addressing L-ASNase immunogenicity is crucial for optimizing treatment outcomes and minimizing adverse effects in leukemia patients receiving this therapy.

The interplay between RBPs, mRNA editing, pyroptosis, and the impact of these factors on AML is an intriguing area of research. Pyroptosis, a form of programmed cell death, modulates the immune response in acute myeloid leukemia (AML) cells. Identifying pyroptosis-related RBP genes and their potential prognostic value in patients with AML could provide critical insights into disease progression and therapeutic strategies. Bin et al. used the Gene expression omnibus (GEO) database to identify pyroptosis-RBP-related differentially expressed genes (PRBP-DEGs) and offer a comprehensive understanding of the interplay between pyroptosis, RNA-binding proteins, and AML prognosis. The established risk model and nomogram hold promise for improving the prognostic accuracy and can guide potential therapeutic strategies for AML.

In the tumor-microenvironment (TME), fatty acid metabolism (FAM) affects tumor cell behavior, interactions with neighboring and immune cells, and extracellular matrix. The complexities of FAM’s influence of FAM on AML in TME is not well understood. Ye et al.'s investigation of scRNA-Seq and bulk transcriptome data on AML patients to explore the association between FAM, TME, and patient outcomes is a key advancement in understanding AML biology and therapeutic opportunities. Elevated FAM-related genes in leukemic stem cells suggest metabolic characteristics driving leukemia progression. PLA2G4A, a highly expressed FAM gene, is linked to poor prognosis in AML, and its targeting enhances NK Cell-mediated Immunosurveillance. This study provides insights into FAM, TME, and immune surveillance in AML, offering potential targeted therapies and personalized interventions to improved the patient outcomes.

Examination of the effects of tyrosine kinase inhibitors (TKI) on cellular metabolism is crucial for improving treatment outcomes in chronic myeloid leukemia (CML) and Philadelphia chromosome-positive acute lymphoblastic leukemia (Ph + ALL) and reducing side effects in pediatric patients. Although TKIs have improved prognosis, challenges like drug resistance, off-target effects, and drug tolerance can impede efficacy. Li et al. highlighted the significance of alterations in glucose, lipid, and amino acid metabolism in influencing treatment responses and drug resistance during TKI therapy in children with Ph+ leukemia. These metabolic changes can affect drug sensitivity and resistance, potentially affects TKI efficacy in targeting leukemia cells.

A study by Zhou et al. on aplastic anemia in children used single-cell RNA sequencing to identify new gene expression patterns and cell subsets, revealing significant findings related to metabolic changes, including genes such as NENF, INPP4B, AKR1C3, and CHST2, which play crucial roles in neurotrophic support, phosphoinositide signaling, steroid and prostaglandin metabolism, and glycosaminoglycan biosynthesis.

FLT3 mutations, including ITD and TKD, are common in AML and affect patient prognosis. FLT3-ITD mutations have been extensively studied due to their adverse prognostic impact on AML. A meta-analysis by Li et al. on FLT3-TKD mutations in AML showed intriguing differences in prognosis between Asian and Caucasian populations. The presence of FLT3-TKD mutations may have a favorable prognosis for DFS and OS in Asian AML patients, whereas in Caucasians, these mutations are linked to an adverse prognosis for DFS. However, caution is advised when interpreting DFS results in Caucasians due to observed heterogeneity among studies.

Transcriptome analysis is crucial for understanding the molecular mechanisms of leukemia, especially mutations in SHP2 that affect signaling pathways involved in cell growth and survival. Zhao et al. conducted transcriptome profiling and identified 2443 and 2273 differentially expressed genes in HCD-57 cells expressing SHP2 mutants compared to parental cells, revealing the impact of mutant SHP2 gene expression.

Research on metabolic reprogramming in hematological malignancies has provided valuable insights, deepening our understanding of cellular metabolic mechanisms and identifying potential treatment targets. Further validation and exploration of these targets is necessary to ensure their efficacy and safety. Continued research is crucial to translate these findings into clinically relevant treatments that could improve the management and outcomes of hematological malignancies.

## Author contributions

ZL: Writing – original draft, Writing – review & editing: JY: Writing – review & editing, Writing – original draft, Supervision, Software. ZZ: Writing – review & editing, Writing – original draft. HS: Writing – review & editing, Writing – original draft. MK: Writing – review & editing, Writing – original draft, Supervision, Resources, Project administration, Funding acquisition, Conceptualization.

